# A comparative analysis of cervical cancer prevention between Nigeria and Nordic countries that have experienced a decline in cervical cancer incidence

**DOI:** 10.1093/inthealth/ihaa062

**Published:** 2020-09-30

**Authors:** Helen I Anyasi, Anna M Foss

**Affiliations:** Prevention Unit, SIDHAS Project, FHI360, No. 8, Yedseram Street, Maitama| P.M.B. 44, Abuja, Nigeria; Department of Global Health and Development, LSHTM, 15-17 Tavistock Place, London, WC1H 9SH, United Kingdom

**Keywords:** cervical cancer, Nigeria, prevention and control, public health systems research, review, Scandinavian and Nordic countries

## Abstract

**Background:**

A modelling analysis carried out in 2014 suggested that, without cervical cancer screening programmes, the incidence of cervical cancer in Denmark, Finland, Norway and Sweden would have been as high as that in some low- and middle-income countries. We compare programme strategies between Nigeria and these Nordic countries and develop translatable recommendations.

**Methods:**

A literature review using a systematic approach through Medline, Popline, Global Health, CINAHL PLUS, Cochrane Library, EMBASE, Google Scholar, Africa Wide and WHO databases was conducted.

**Results:**

Fifteen journal articles and two grey literature reports met our criteria. Six descriptive studies from Nigeria noted that services in Nigeria were mainly provided in urban secondary/tertiary facilities and that uptake was low even where screening was free. Trials in Nigeria and Sweden noted that subsidies and free programmes alone did not improve uptake; a Danish trial demonstrated that reminders and invitations issued by general practitioners improved participation.

**Conclusion:**

Free screening programmes are important but should also consider incentivisation of treatment when needed and demand creation among health workers. Additionally, effective monitoring and evaluation of programme data are key to improving and maintaining quality. More broadly, we suggest that Nigeria can build success through stakeholder-led implementation of well-defined policies with national consensus to ensure coordination and sustainability.

## Introduction

Cervical cancer is a disease of great public health interest because it is easily preventable and primarily affects women aged 15–44 y, an age group within which women make great social and economic contributions.^1^ The main cause of cervical cancer is persistent infection with HPV, which is sexually transmitted; precancerous lesions develop over 5–10 y, from which invasive cervical cancer can develop decades later.^2^ In Nigeria, 70 327 deaths in women were attributed to cancer, with cervical cancer causing 14.8% of those deaths in 2018, making it the second most common cancer after breast cancer.^3^ A frequent occurrence in Nigeria is a trend of late presentation and diagnosis at advanced stages of the disease leading to poor prognosis.^4^^,^^5^

About 118 million women have been immunised against HPV, of which only 1% are from low- to middle-income countries,^6^ with numerous barriers reported.^7^^,^^8^

In some developed countries a marked reduction in cervical cancer incidence has been noted through organised screening programmes, with adoption of national screening algorithms and guidelines.^9^^–^^14^ Time trends were used to ascertain mortality from cervical cancer following the advent of screening programmes since the early 1950s in Denmark, Finland, Iceland, Norway and Sweden; the authors reported that in these five countries mortality rates fell by 10–80%.^15^ More recently, a modelling analysis of cervical cancer screening programmes in four countries in the Nordic region (Denmark, Finland, Norway and Sweden), using ad hoc-refined age-period-cohort models, suggested that cervical cancer incidence rates in the Nordic countries would ‘have been otherwise comparable to the highest incidence rates currently detected in low-income countries’ if not for their cancer prevention programmes.^10^

Existing studies in the broader literature relates cervical cancer screening gaps to high incidence in Nigeria, highlighting determinants of low screening rates such as poor awareness of screening services^16^^,^^17^ and psychosocial factors like poor risk perception and fear of positive results.^18^^,^^19^ In another study, screening gaps were attributed to demand-side barriers like cost and the non-availability of local screening services.^20^ These, and similar studies,^16^^–^^19^^,^^21^^,^^22^ provide a basis for proposing solutions and charting a course forward towards progress.

## Aim and Objectives

Our aim is to assess the current state of cervical cancer prevention strategies in Nigeria and compare this with evidence-based interventions that are/were implemented in the four Nordic countries of interest, namely, Denmark, Finland, Norway and Sweden, to highlight relevant lessons learned for the Nigerian context.

The specific objectives are to:

1. Ascertain how cervical cancer prevention services are/were implemented in Nigeria and across the four selected Nordic countries by considering method of delivery (opportunistic vs organised) and the availability of services at rural and urban health facilities.
2. Assess the effect of targeted interventions on women's uptake of services.
3. Investigate potential factors said to be responsible for the decline in cervical cancer incidence in these Nordic countries and to seek relevant lessons learned for implementation in Nigeria, discussing context-specific limitations to generalisability.

Through this review we intend to broaden the prior knowledge base by seeking how interventions are currently implemented across all these countries and what was/is being done to improve implementation. In looking holistically at these systems, we will consider both demand- and supply-side factors that help or hinder intervention strategies. In addition, our review will add to the earlier work of others through our novel cross-continent comparison, in which we seek common themes arising in the Nordic countries and explore the feasibility of these to serve as a model for Nigeria or to influence future innovations.

## Materials and Methods

Our review follows the PRISMA guidelines and checklist (see Appendix 1 in the Supplementary Data file).^23^

### Literature searches

Published peer-reviewed journal articles and studies on cervical cancer prevention and control programmes in the selected countries were obtained by conducting database searches in Ovid Medline, Popline, Global Health, CINAHL PLUS, Cochrane Library and EMBASE. Searches were undertaken in all the databases using a combination of MeSH terms and free text. Appendix 2 in the Supplementary Data file shows the search concepts and synonyms that were then joined with Boolean operators to form the following search string of concepts (into which their synonyms were added): ‘Cervical Cancer* AND Cancer screen* AND (Nigeria OR Nordic Countries)’. Grey literature was sought using Google Scholar, Africa Wide and WHO databases. To refine searches in Google, keywords were used in addition to the URL of interest (e.g. ‘cervical cancer screening Nigeria who.int’).

Articles were limited to those written in English and/or translated to English. Date limits were set to start at the year 2000. The dates of initial database searches for papers reviewed are as follows: Africa Wide on 26 June 2018, PopLine, MedLine and Global Health on 25 July 2018, CINAHL PLUS, Cochrane Library and Embase on 26 July 2018 and Google Search on 28 July 2018. To ensure no more recent publication was missed, database searches were repeated on the following dates: MedLine on 21 October 2019, Africa Wide, Global Health, CINAHL PLUS, Cochrane Library and Embase and Google Scholar on 5 November 2019. (Popline was retired on 1 September 2019, so we were unable to rerun searches there.) The complete search strategies are available in Appendix 3 of the Supplementary Data file.

### Limits and selection criteria

Studies were included only if they had information about cervical cancer prevention programmes administered by governmental or non-governmental institutions, private institutions or international organisations in the countries of interest, either as a stand-alone programme or as part of an integrated package of care. Only secondary prevention is being considered in this review so the methods for early cancer diagnosis were limited to visual inspection with acetic acid, visual inspection with lugol iodine, test and treat, HPV testing and pap smear.

### Data extraction and quality assessment

Relevant data from the selected studies were extracted into a table to facilitate the review process. Studies were assessed for quality guided by NIH quality assessment tools (QAT) for controlled intervention, observational and cohort studies.^24^ The papers were appraised on whether the aims were clearly and logically specified, the methodology was appropriate to the aims and if the study populations were well defined. Studies were assessed based on the number of affirmative responses to the QAT criteria and classified into ratings of good, fair or poor. The cut-off point for the ratings are described in National Heart, Lung, and Blood Institute’s quality assessment tools.^24^

For the WHO reports obtained from grey literature searches, the quality was authenticated using The Sheridan Libraries at Johns Hopkins University open source guide and template for evaluating data sources from the internet using the acronym CRAAP: Is it Current enough, Relevance, Authority? What makes me trust the information, Accuracy, and Purpose?^25^ The parameter ‘Relevance’ was removed as the inclusion/exclusion criteria already accounts for this. The sample data extraction form, critical appraisal tools and tables for all the academic and grey literature can be found in Supplementary Data file (Appendices 4–7)

## Results

### Summary of articles and reports included in the review

**Figure 1.**
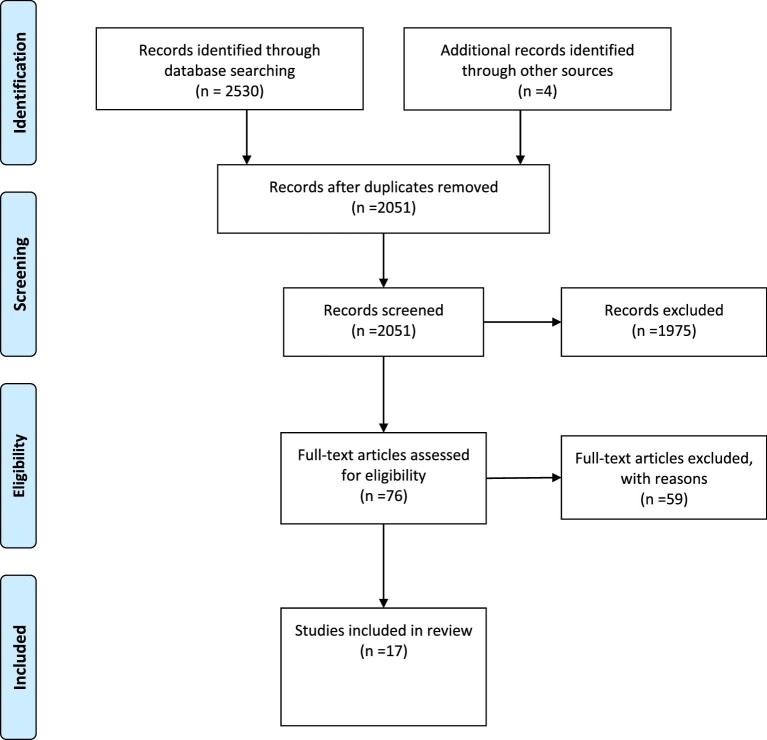
PRISMA^23^ diagram.

A total of 2051 non-duplicated publications were identified for screening, with 1975 being screened out through title or abstract alone. The full texts for the other 76 articles were obtained and reviewed. Of the 76 full-text publications assessed for eligibility, we excluded 59 publications for one or both of the following reasons: (1) little or no information provided about the implementation of cervical cancer prevention programmes; (2) the focus of the paper was cervical cancer prevention via primary prevention (HPV vaccination) rather than secondary prevention. This process resulted in 17 publications that met the selection criteria. Figure 1,^23^ shows details of records identified, screened, included and excluded.

Fifteen journal articles and two grey literature items were included in the review having met the selection criteria. These articles were diverse with six descriptive studies^26^^–^^31^ (including one that was population-based^31^), one randomised experimental study,^32^ two population-based cluster randomised trials,^33^^,^^34^ one cohort study,^10^ five articles involving secondary data analyses^35^^–^^39^ and two reports.^40^^,^^41^ Six of the academic studies published in journals were set in Nigeria and nine were from the Nordic countries of interest (Table 1). The WHO report describes the outcomes of cervical cancer pilot projects in six African countries including Nigeria.^40^ The second report is on the status of implementation of cancer-screening programmes in the European Union, of which three of the four countries of interest were discussed.^41^

**Table 1.** Summary of study characteristics for articles/reports included in the review

Authors	Study characteristics	Outcomes reported by authors	Authors’ conclusions
Journal articles in Nigerian settings (including reviews)			
Bassey et al (2008)^30^	Descriptive study	Poor uptake of services	There are no organised cervical cancer screening programmes although there are facilities for cytology in some Nigerian hospitals, which serve a limited number of women
Chukwuali et al (2003)^28^	Descriptive study	Poor uptake of services: only 815 women participated in the highly subsidised screening service in Enugu over a 10-y period	Due to reasons such as poor awareness and sociocultural barriers, subsidised cervical cancer screening was not adequately utilised
Adepoju et al (2016)^26^	Descriptive study	Challenges to accessing cervical cancer screening: disparity of location in favour of urban tertiary facilities, low risk perception and logistical issues in rural areas	Since most participants were urban-based, there is need to decentralise cancer of cervix screening through mobile clinics and establishment of screening centres in the rural areas
Obi et al (2007)^27^	Descriptive study	Poor participation as <1% (932 women) of target population participated	It was not enough to provide cervical cancer screening services but there is a need to follow up these services by sustained awareness campaigns and motivation of healthcare providers to offer appropriate information to patients
Nnadi et al (2016)^29^	Descriptive study	Participation was extremely poor compared with similar studies conducted in other parts of the country. Indication for cervical screening was mostly symptom-based referrals from facilities without screening services within and outside the state	Only through formulation and implementation of an organised national screening programme (while maximising opportunistic screening in the interim) can screening be performed more effectively and efficiently
Okeke et al (2012)^32^	Randomized experimental study	Barriers to access include distance and travel costs; women who were randomly selected to receive the conditional cancer treatment subsidy were about 4% more likely to accept cervical cancer screening	The optimal set of subsidies should include treatment subsidies (if the client is screened positive) in addition to screening price subsidies
Alfonzo et al (2016)^33^	Population-based randomized controlled trial	Participation was not affected by the absence or presence of a fee	Other strategies could be employed in socially disadvantaged urban districts as abolishing fees did not increase attendance in the short term
Journal articles in Nordic settings (including reviews)			
Jensen et al^34^ (2009)	Cluster randomized controlled trial	Improved participation and improved coverage when women were targeted with invitations and enhancement of GPs’ attention to cervical cancer programmes in Denmark	Using a special targeted invitation to non-attendees combined with increasing GPs’ attention to the programmes could improve women's participation and increase coverage of cervical cancer screening
Elfstrom et al (2016)^31^	Population-based descriptive study	Analysing key quality indicators formed the basis for quality improvement of the organised cervical screening programmes in Sweden	Regular registry-based monitoring and evaluation of quality indicators can provide an evidence base for prioritisation of improvement strategies
Vaccarella et al (2016)^44^	Cohort study	In the absence of screening, incidence rates for 2006–2010 in Nordic countries would have been fivefold higher than observed rates	The organised screening programs in these four Nordic countries have resulted in the low incidence of cervical cancer
Dillner (2000)^35^	Review article	Cervical cancer screening in Sweden is heterogeneous in quality, i.e. some counties practise organised screening and others are opportunistic	More studies need to be conducted to assess the effect of organised screening vs spontaneous screening on cervical cancer mortality
Hortlund et al (2018)^39^	Research article	2278 000 cervical samples collected in Sweden in 2014–2016 with 69% coming from the organised screening programme. Screening coverage was 82% (an average of 71–92% within counties); cervical cancer showed an increasing trend	Key quality indicators such as population coverage and follow-up rates were stable or improving, but nevertheless there was a cervical cancer increase suggesting that current efforts for measuring and reporting quality indicators are insufficient
Anttila et al (2000)^36^	Review article	Incidence of cervical cancer has decreased in Finland and this is attributed to organised screening activities	The 30-y-old organised screening programmes have resulted in a decrease of >70% cervical cancer incidence and a reduction in cervical cancer mortality
Nygård et al^38^ (2002)	Review article	The Norwegian coordinated programme introduced in 1995 collected a total of 4744 967 pap smears from >1.4 million women aged 25–69 y recommended to have a conventional pap smear every 3 y	The screening programme provides a low-cost way to increase coverage as the number of women who had a pap smear was higher after implementation of the coordinated programme of women aged 23–59 y
Bigaard et al (2000)^37^	Review article	Danish screening programmes had good coverage as a total of 650 000 smears were taken annually, which corresponds to screening of all Danish women aged 25–59 y on average every second year, even although the guidelines recommend screening every third year. There was a decrease in incidence from 15.3 per 100 000 women during 1987–1992 to 12.9 per 100 000 women during 1993–1995	Organised screening has a better preventive effect than opportunistic screening; they recommend a move towards a longer screening interval than the 3-y interval currently practised
Grey literature			
WHO (2012)^40^	Project report	Observation of poor uptake and coverage in Nigeria	There is a need for effective monitoring and evaluation system to track key performance indicators such as coverage and incidence
Ponti et al (2017)^41^	Meeting report	The Finnish programme has proven to be very effective in reducing the incidence of and mortality from cervical cancer	The Finnish cervical cancer screening programme is an example of a cost-effective way to run an organised programme

### Quality assessment

Fifteen journal articles were assessed using the QAT criteria. Of the articles selected, two were rated as ‘low’ because (1) small sample sizes were selected without clear description of their methodological design, which restricted generalisability to the general population, (2) we were unable to confirm if biases and confounding factors were sufficiently considered from the information provided and (3) we were not able to ascertain ethical consideration from the information provided.^27^^,^^30^ Two papers were rated as ‘fair’ because the methodological design was not adequately described to give information on sample selection and study duration.^26^^,^^28^ None of these papers were excluded from the review but these limitations should be kept in mind when interpreting the findings.

### Cervical cancer screening in Nigeria and the Nordic countries of interest: method of delivery and availability of services at rural and urban health facilities

#### Method of delivery

In both Nigerian and Nordic settings, the authors attempt to explain achievements (or gaps) in the screening programmes by considering the nature of screening (i.e. opportunistic vs organized). The International Agency for Research on Cancer defines opportunistic screening as ‘early detection of cancer performed in a diagnostic or clinical context or performed often in a prophylactic purpose’.^41^ For cervical cancer-screening programmes to be considered organised they must have the following: (1) clear policies that specify the target population, the type of screening tests and screening intervals; (2) public funding; (3) a system for inviting women for screening; (4) a team responsible for overseeing programme implementation; and (5) structures to ensure quality improvement.^41^ This definition clarifies findings across the literature and is helpful to categorise implementation methods across settings.

From the literature it is noted that screening is largely opportunistic in the Nigerian setting.^26^^–^^29^ The study authors noted that women's participation was poor and proffered different reasons as to why service uptake was low. Reasons included poor demand creation and weak information dissemination,^26^^,^^27^^,^^29^ low referral rates from general practitioners (GPs),^27^ low risk perception, fear of getting a positive result^26^^,^^28^^,^^32^ and cultural barriers stemming from the nature of the screening procedure, which involves a pelvic exam.^32^ There is consensus among the authors that, although there is no nationally organised programme in Nigeria, opportunistic screening needs to be maximised.

Table 2 provides information on cervical cancer screening programme activities in the four Nordic countries of interest.

**Table 2.** Information on the four Nordic countries programme implementation culled from the 2017 WHO EU Meeting Report and Vaccarella et al. (2014) study

General information				Programme organisation					Programme monitoring and quality assurance	
Country	Description of national screening activities	Target age (y)	Screening interval (y)	Screening activities	Is there a national screening policy documented as a law or an official recommendation?	Is there a team responsible for implementing the policy?	Are the screening tests provided free of charge?	Does the programme issue individual invitations through the screening registries?	Is there a team responsible for quality assurance?	Are screening data collected, linked to screening registries with programme performance reports published?
Finland	1963	30–64^1^	5	National, population-based	OR	✓	✓	✓	✓	✓
Sweden	1967	23–60	3 (23–50); 5 (51–60)	National, population-based	OR	✓	✓	✓	✓	✓
Norway	1995	25–69	3	National, combination of opportunistic and organised activities	OR	✓	✗	✓	✓	✓
Denmark	1967	23–59 (HPV test: 60–65)	3 (23–59); 5 (60–64)	Regional until 1996; now national and population-based	OR	✓	✓	✓	✓	✓

✗ = NO;

✓ = YES;

^1^Some municipalities target women below 30 years and above 60 years

^3^OR=Official recommendation

It is noted that Denmark, Sweden and Finland implement organised screening.^10^^,^^41^ Norway, however, implements both opportunistic and organised screening activities at a fee.^10^

Common denominators in the four Nordic screening programmes are:^10^^,^^31^

1. Long-term population-based screening;
2. Specified target populations with country-specific screening intervals;
3. Invitations to eligible women through the screening registries;
4. Quality improvement processes that are overseen by working groups or similar national bodies;
5. Regular data collection with well-established national databases and national cancer registries.

Sweden and Finland have the longest running cervical cancer programmes in the world, both of which started in the 1960s.^41^ Sweden expanded organised screening to 69% with overall screening coverage (including opportunistic screening) at 82% after intensifying efforts to issue invitations for screening.^35^^,^^39^ In Denmark, it was noted that organised screening had a more preventive effect compared with opportunistic screening as they attribute a 3% decrease in incidence from the late 1980s to the early 1990s to the introduction of organised screening over that period.^37^ The Finnish organised programme is said to have contributed to a 70–80% decrease in the age-adjusted cervical cancer incidence,^42^ which was also the case in Norway, where a 22% decrease was noted after changes were made to improve coordination in their screening programme.^38^ Additionally, both the Finnish and the Norwegian organised screening programmes were reported to be cheaper and more cost-effective.^36^^,^^38^

#### Availability of services at rural and urban health facilities

Screening services in Nigeria are provided mainly in urban tertiary and secondary facilities.^26^^,^^32^ Most of the screening that occurs is due to the presence of symptoms suggestive of cervical abnormality.^29^ In Sweden and Finland, trained midwives collect samples at local maternity centres and send them to reference laboratorys for assay.^35^^,^^42^ In Norway and Denmark, when women receive their invitation letters for screening, it states that they are due for a pap smear and should visit their current GP or gynaecologist.^37^^,^^38^

### Effect of targeted interventions on participation and service uptake

Six articles focused on the effect of interventions on women's uptake of screening services, with the locations of these studies being in Denmark, Sweden and Nigeria (Table 3).

The trial in Sweden reported that there was no significant difference between the control group that was required to pay a fee for screening and the intervention group that had the fee abolished.^33^ The authors suggest that other interventions for improving participation might be more effective, such as those that were offered to both the intervention and control groups in this study (namely, targeted individualised communication and education, rescheduling of screening online and annual re-invitations sent to non-attendees), which were thought to have led to the observed increase in attendance noted across both groups during the study period.^33^

The Danish study authors concluded that screening coverage and participation can be increased by using a special targeted invitation to non-attendees combined with increasing GPs’ attention to the programme.^34^

Within Nigerian settings, researchers studied strategies such as free screening, cost subsidies and other incentives to improve participation and coverage as part of the intervention.^26^^,^^27^^,^^30^ For example, Okeke et al. used a lottery system to determine who would get free screening and possible treatment if screened positive; they found that women who received cancer treatment subsidies were four percentage points more likely to take up screening than those in the control group (a 30% relative increase).

**Table 3.** Description of targeted interventions across three countries

Country; paper	Intervention	Findings
Sweden; Alfonzo (2016)^33^	Women who were to be invited for screening were randomised 1:1, to receive an invitation either stating that the test was free (intervention group) or that it cost 100 SEK (control group)	Researchers discovered no significant differences between women who were charged and those offered free screening (RR 0.93; 95% CI 0.85 to 1.02). There were also no variances within the districts, age and attendance after the most recent previous invitation or previous experience of smear-taking
Denmark; Jensen (2009)^34^	Normal letter at 3 y intervals to all women + a specific targeted letter to non-attendees + GP received visit by facilitators/advocates	The decline in non-attendees was 0.87% after 9 mo in favour of the intervention. A difference of 0.94% in the change of coverage rate was observed at 6 mo, which increased to 1.97% at 9 mo in favour of the intervention
Nigeria; Okeke (2013)^32^	1. Scratch cards offered to women to provide screening at N0, N50 (US$0.33) and N100 (US$0.66)	Women who were randomly selected to receive the conditional cancer treatment subsidy were about 4% more likely to accept cervical cancer screening
	2. Lottery tickets for treatment subsidies	
Nigeria; Obi (2007)^27^	The intervention was described as a ‘highly subsidised’ screening programme in Enugu. The nature of the subsidy was not described	Authors report poor participation as <1% (932 women) of the target population were reached
Nigeria; Adepoju (2016)^26^	Free cervical cancer screening programme sponsored by the Osun State government	Uptake of cervical cancer screening was low
Nigeria; Bassey (2008)^30^	In the period under study, screening was free of charge at three selected hospitals in Uyo	The study reports poor participation of the target population as only 332 women participated in the 5 y when free monthly screening was offered

All the other studies that specifically addressed user fees found that there was no significant change in uptake either through removal of the fee or by reducing the amount, as observed in the randomised intervention in Sweden.

The collective evidence supports the conclusion of Okeke et al. that the optimal set of subsidies to increase service uptake in developing countries must include subsidies towards treatment costs in addition to price subsidies for screening.^32^

## Discussion

### Summary of findings

This review aimed to explore the current state of cervical cancer prevention strategies in Nigeria and contrast this with evidence-based interventions that were implemented in some Nordic countries that have experienced a decline in cervical cancer incidence. We sought to ascertain methods of implementation, the impact of interventions designed to increase screening uptake and other factors limiting or contributing to success.

We find in this review that cervical screening services in Nigeria are mainly located in urban secondary and tertiary health centres. This contrasts sharply with the service delivery model used in Sweden and Finland, where healthcare workers in local maternity centres have the capacity to collect pap smears. It may also explain the good coverage rates, especially in Sweden where average coverage is 82% across all counties, although we cannot ascertain which counties are in rural areas.

We also found that the implementation of screening in Nigeria is opportunistic with evidence suggesting that opportunities are not fully maximised by caregivers.^20^^,^^28^^,^^29^ When reviewing the evidence, we found that organised screening was recommended over opportunistic screening because it was the deemed to be a more successful means of achieving full coverage. It is also touted to be cheaper and more cost-effective in the long run. In three of the four Nordic countries reviewed, mostly it is organised screening that is implemented, with the fourth country (Norway) implementing both organised and opportunistic programmes. Still, variations exist in implementation, with areas of overlap between opportunistic and organised strategies in all four countries.^43^ It is important to note that these countries all made changes to move towards organised screening; for example, in Sweden, active steps were taken to homogenise their programme, which was believed to improve coverage.^35^ Other advantages of organised screening include reduced healthcare costs, universal coverage and social inequalities, and improved registration and monitoring.^41^ However, opportunistic testing leads more directly to treatment where disease is found; also planning, preparation and completion of an organised implementation process requires a long timeframe and extensive resources.^41^ Bearing this in mind, it is important to acknowledge what is possible in each setting, given differences in resources and health system structures.

Studies specifically addressing user fees found that there was no significant change in uptake attributed to either abolishing or reducing a fee.

The four Nordic countries of interest use an invitational method for encouraging women to attend testing. Women are expected to have at least 9–12 screening tests in their lifetimes, depending on the screening interval of 3–5 y. It is noted that coverage is better in countries that invite all women targeted, and not only women who did not attend screening.^41^

### Strengths and limitations

This review was conducted using a systematic approach: data were extracted by one reviewer (HIA) while a senior researcher (AMF) had oversight over limits and selection criteria. This review greatly benefitted from good coverage of databases provided by the LSHTM Library. Another strength was our search for grey literature in addition to academic literature. The start date limit was set to the year 2000, to keep the search focused on the last 2 decades of cervical cancer screening programmes, although this was likely a key reason why more articles about the early programmes in Nordic countries were not included in the review. Due to translation resource limitations, we only searched for papers in English, which is likely to have had a negligible impact in terms of the Nigerian studies since English is the lingua franca in Nigeria, but we may have missed papers written in Finnish, Danish, Norwegian or Swedish. Reassuringly, our strategy and criteria led to the inclusion of the expected landmark studies related to the subject matter.^32^^,^^44^

Initial database searches were undertaken in June/July 2018. To ensure that no more recent publications were missed, we re-ran searches in October/November 2019 to yield the most up-to-date data. Another key strength was that grey literature was included in the search and identified additional resources for the review.

We synthesised the literature successfully despite the heterogeneity of the populations, study designs, outcome measures and findings. Few studies provided population-level data or cervical cancer statistics collected at central registries, which limited the extent of more complex analyses of incidence or prevalence rates that could be conducted. We also had difficulty ascertaining the coverage and impact of screening programmes in the Nigerian setting. Although there were some quality issues with four of the studies,^26^^–^^28^^,^^30^ the data extracted from these were in alignment with those extracted from other sources.

We noted differences in what countries considered nationwide population-based screening; there were overlaps in opportunistic regional screening and national organised screening in the Nordic countries. Despite these limitations clear themes arose and these are developed below as recommendations.

### Recommendations

The Nordic cervical screening programmes have spanned 50 y and survived changes in government, shifts in sociocultural and economic ebbs and flows that occur naturally over time. Our findings suggest that the Nordic programmes have thrived because national screening policies were enforced by law or by official recommendation with vital stakeholders at the helm of overseeing policy transformed to practice.^31^^,^^35^ Sustainability and continuity are inherent when programmes are country-led and driven by key actors and stakeholders rather than time-bound, donor-driven activities, as commonly found in Nigeria,^45^ which may end upon a stipulated project close-out date.

The Nigerian National Cancer Control Policy, developed for implementation from 2018 to 2022, highlights breast cancer and cervical cancer as the cause of 50% of all cancer deaths in country.^46^ It also notes ‘the absence of well-coordinated national screening programmes…significantly contributing to late presentation of most cancer patients’.^46^ We, therefore, recommend an evaluation and review of the current cancer policy implementation in Nigeria, with an aim to prioritise the top cancers that cause mortality and institute a working group composed of key state actors and stakeholders to oversee policy implementation and evaluate progress.

Findings suggest the need, across both settings, for an effective monitoring and evaluation system to track key performance indicators such as coverage and incidence.^39^ Also, the detailed and consistent monitoring and evaluation of programme quality improvement processes provides a reliable evidence base for prioritisation and evaluation of improvement strategies. Therefore, we recommend not just a system for monitoring and evaluation of programme data with key performance indicators to track programme impact but also a monitoring and evaluation of programme quality improvement processes to inform activities geared towards prioritisation of improvements.

In Nigeria, where major health services are centralised in urban tertiary facilities, a foremost concern should be decentralisation of services. The Nigerian National Cancer 2018–2022 control plan has, as one of its objectives, to ensure that 40% of all healthcare facilities (at primary, secondary and tertiary levels) are strengthened through increased institutional capacity to deliver cancer screening/early detection.^46^ This idea is not original as it is one of the hallmarks of success in the Nigerian HIV programme; there was a move from a vertical to a more integrated, provider-initiated service provision^47^^,^^48^ with decentralisation from tertiary to secondary and primary health facilities.^49^ This can serve as a blueprint for the Nigerian cervical cancer programme.

Although all the Nordic countries moved to homogenise their programmes to be organised, ‘opportunistic testing leads more directly to treatment where disease is found; also planning, preparation and completion of an organised implementation process requires a long timeframe and extensive resources’.^41^ Therefore, we acknowledge the differences in resources and health system structures in these two settings and recommend that opportunistic screening be maximised in Nigeria by engaging health workers to offer services, active referrals and client education by caregivers, while considering other effective ways to improve screening coverage. It is also important to include the ‘screen and treat’ option, where cytology-based tests are not possible, as treatment can be provided in a single visit where necessary. It is also believed to be a more low-cost and low-technology method, especially when considering implemention in rural settings.^40^

The evidence suggests that reducing or removing fees alone is insufficient to improve women's participation in cervical cancer screening initiatives in Nigeria, yet there are claims that the biggest barrier to healthcare access in Nigeria is finance,^20^ hence we cannot simply disregard the usefulness of interventions that provide screening free of charge or at a subsidised rate. Prioritised programmes in Nigeria, like those for Malaria and HIV that are heavily subsidised by the government and donors, have achieved appreciable levels of success owing to this support.^50^^–^^53^ Evidence from the Swedish trial^33^ included in our review shows that health education and individualised communication can improve screening coverage. This is also supported by a review that examined health education interventions across developed and developing countries, showing that they boosted screening coverage.^54^ Therefore, we do not discount the alleviating effect of subsidy; rather, we suggest that, in addition to offering screening free of charge or offering subsidies for screening alone, consideration should be given to a more complex package that may include health education interventions, individualised communication, treatment subsidies in the event of a positive screening outcome^32^ and other means of incentivisation such as payment vouchers or prepayment schemes.^20^

The invitational system used in the four Nordic countries, to invite all women targeted, requires cancer screening registries with information databases. This may not currently be feasible in the Nigerian setting, owing to lack of cancer registries, but there are existing structures that can be leveraged, such as the biannual Maternal, Newborn, Child Health Week, which is used to reach large numbers of the women in this defined target group. Coverage can be ascertained using available and extrapolated data to estimate population size. We also suggest initial pilots that can lead to scale-up so that lessons learnt can be documented and improvements made iteratively during expansion. This was the case in Finland, where pilots first started within the area of three municipalities in 1963 and then were extended within a few years to most parts of the country.^36^

While HPV vaccination will be important in further reducing incidence, beyond screening alone, there are numerous barriers to uptake as, to date, the HPV immunisation rate is only 1% in low- and middle-income countries.^6^ Hence, we see our recommendations as robust for the short to medium term.

### Conclusions

A more successful approach for Nigeria, in addition to providing subsidised or free screening programmes, would include country-led action driven by state actors and key stakeholders to ensure continuity and sustainability of targeted interventions. Key interventions geared towards improving uptake could comprise incentivisation of treatment if screened positive and active engagement of health workers for demand creation. Additionally, well-defined policies that guide service provision are needed, alongside a population-based approach to implementation (including rural populations), and an effective monitoring and evaluation of programme data for quality improvement of programme delivery with a plan for continuous research and development in cervical cancer programming.

## Supplementary Material

ihaa062_Supplemental_File

## Data Availability

All the data used are from the literature and so are in the public domain.
