# Supplementary material for: A comparative analysis of cervical cancer prevention between Nigeria and Nordic countries that have experienced a decline in cervical cancer incidence
Source: Int Health. 2020 Sep 30;13(4):307–17. doi: 10.1093/inthealth/ihaa062 (PMC8253993; doi:10.1093/inthealth/ihaa062)
Supplement: ihaa062_Supplemental_File [file ihaa062_supplemental_file.docx]

APPENDIX 1: PRISMA CHECKLIST

| **TITLE** | | | | |  |
| --- | --- | --- | --- | --- | --- |
| Title | | 1 | Identify the report as a systematic review, meta-analysis, or both. | | 2 |
| **ABSTRACT** | | | | |  |
| Structured summary | | 2 | Provide a structured summary including, as applicable: background; objectives; data sources; study eligibility criteria, participants, and interventions; study appraisal and synthesis methods; results; limitations; conclusions and implications of key findings; systematic review registration number. | | 2 |
| **INTRODUCTION** | | | | |  |
| Rationale | | 3 | Describe the rationale for the review in the context of what is already known. | | 4 |
| Objectives | | 4 | Provide an explicit statement of questions being addressed with reference to participants, interventions, comparisons, outcomes, and study design (PICOS). | | 5 |
| **METHODS** | | | | |  |
| Protocol and registration | | 5 | Indicate if a review protocol exists, if and where it can be accessed (e.g., Web address), and, if available, provide registration information including registration number. | | N/A |
| Eligibility criteria | | 6 | Specify study characteristics (e.g., PICOS, length of follow-up) and report characteristics (e.g., years considered, language, publication status) used as criteria for eligibility, giving rationale. | | 6-7 |
| Information sources | | 7 | Describe all information sources (e.g., databases with dates of coverage, contact with study authors to identify additional studies) in the search and date last searched. | | 6 |
| Search | | 8 | Present full electronic search strategy for at least one database, including any limits used, such that it could be repeated. | | Appendix 2 |
| Study selection | | 9 | State the process for selecting studies (i.e., screening, eligibility, included in systematic review, and, if applicable, included in the meta-analysis). | | 8 |
| Data collection process | | 10 | Describe method of data extraction from reports (e.g., piloted forms, independently, in duplicate) and any processes for obtaining and confirming data from investigators. | | 7 |
| Data items | | 11 | List and define all variables for which data were sought (e.g., PICOS, funding sources) and any assumptions and simplifications made. | | Table 2 |
| Risk of bias in individual studies | | 12 | Describe methods used for assessing risk of bias of individual studies (including specification of whether this was done at the study or outcome level), and how this information is to be used in any data synthesis. | | 7 |
| Summary measures | | 13 | State the principal summary measures (e.g., risk ratio, difference in means). | | 13 |
| Synthesis of results | | 14 | Describe the methods of handling data and combining results of studies, if done, including measures of consistency (e.g., I^2^) for each meta-analysis. | | N/A |
| Risk of bias across studies | 15 | | | Specify any assessment of risk of bias that may affect the cumulative evidence (e.g., publication bias, selective reporting within studies). | 15 |
| Additional analyses | 16 | | | Describe methods of additional analyses (e.g., sensitivity or subgroup analyses, meta-regression), if done, indicating which were pre-specified. | N/A |
| **RESULTS** | | | | |  |
| Study selection | 17 | | | Give numbers of studies screened, assessed for eligibility, and included in the review, with reasons for exclusions at each stage, ideally with a flow diagram. | Figure 1 |
| Study characteristics | 18 | | | For each study, present characteristics for which data were extracted (e.g., study size, PICOS, follow-up period) and provide the citations. | Table 1 |
| Risk of bias within studies | 19 | | | Present data on risk of bias of each study and, if available, any outcome level assessment (see item 12). | 7 |
| Results of individual studies | 20 | | | For all outcomes considered (benefits or harms), present, for each study: (a) simple summary data for each intervention group (b) effect estimates and confidence intervals, ideally with a forest plot. | 9-12 |
| Synthesis of results | 21 | | | Present results of each meta-analysis done, including confidence intervals and measures of consistency. | 12 |
| Risk of bias across studies | 22 | | | Present results of any assessment of risk of bias across studies (see Item 15). | 13 |
| Additional analysis | 23 | | | Give results of additional analyses, if done (e.g., sensitivity or subgroup analyses, meta-regression [see Item 16]). | N/A |
| **DISCUSSION** | | | | |  |
| Summary of evidence | 24 | | | Summarize the main findings including the strength of evidence for each main outcome; consider their relevance to key groups (e.g., healthcare providers, users, and policy makers). | 12 |
| Limitations | 25 | | | Discuss limitations at study and outcome level (e.g., risk of bias), and at review-level (e.g., incomplete retrieval of identified research, reporting bias). | 15 |
| Conclusions | 26 | | | Provide a general interpretation of the results in the context of other evidence, and implications for future research. | 17 |
| **FUNDING** | | | | |  |
| Funding | 27 | | | Describe sources of funding for the systematic review and other support (e.g., supply of data); role of funders for the systematic review. | N/A |

**APPENDIX 2: KEY CONCEPTS AND SEARCH TERMS**

| **[Cervical Cancer*](https://leo.lshtm.ac.uk/Print/Form/10122?redacted=true#page=2)** | **[Cancer screen*](https://leo.lshtm.ac.uk/Print/Form/10122?redacted=true#page=2)** | **[Nigeria](https://leo.lshtm.ac.uk/Print/Form/10122?redacted=true#page=2)** | **[Nordic Countries](https://leo.lshtm.ac.uk/Print/Form/10122?redacted=true#page=2)** |
| --- | --- | --- | --- |
| [Cervical Neoplasm* OR Uterine cervical neoplasm* OR Uterine cervical cancer* OR Cancer of the uterine cervix OR Cancer of cervix](https://leo.lshtm.ac.uk/Print/Form/10122?redacted=true#page=2) | [Early cancer detection OR Mass screening OR Cancer screening test* OR Early diagnosis of cancer OR Visual Inspection with Acetic Acid OR HPV testing OR HPV DNA Testing OR Visual Inspection with Lugol Iodine OR VIA OR VILI OR Papanicolaou’s smear OR Pap smear](https://leo.lshtm.ac.uk/Print/Form/10122?redacted=true#page=2) | [Africa, South of the Sahara OR Western Africa OR Federal OR Republic of Nigeria OR Sub-Saharan Africa](https://leo.lshtm.ac.uk/Print/Form/10122?redacted=true#page=2) | [Nordic States OR Nordic region OR Scandinavia* OR Denmark OR Sweden OR Finland OR Norway](https://leo.lshtm.ac.uk/Print/Form/10122?redacted=true#page=2) |

**APPENDIX 3: SEARCH STRATEGY**

Database: Embase Classic+Embase <2000 to 2018 June 25>

Search Strategy:

1 (Cervical Cancer or Cervical Neoplasm* or Uterine cervical neoplasm* or Uterine cervical cancer* or Cancer of the uterine cervix or Cancer of cervix).mp. [mp=title, abstract, heading word, drug trade name, original title, device manufacturer, drug manufacturer, device trade name, keyword, floating subheading word, candidate term word] (59943)

2 (Cancer screen* or "Early cancer detection" or "Mass screening" or "Cancer screening test*" or "Early diagnosis of cancer" or "Visual Inspection with Acetic Acid" or "HPV testing" or "HPV DNA Testing" or "Visual Inspection with Lugol Iodine" or VIA or VILI or "Papanicolaou’s smear" or "Pap smear").mp. [mp=title, abstract, heading word, drug trade name, original title, device manufacturer, drug manufacturer, device trade name, keyword, floating subheading word, candidate term word] (1224521)

3 ("Nigeria" or "Africa, South of the Sahara" or "Western Africa" or "Federal Republic of Nigeria" or "Sub-Saharan Africa").mp. [mp=title, abstract, heading word, drug trade name, original title, device manufacturer, drug manufacturer, device trade name, keyword, floating subheading word, candidate term word] (67190)

4 (Nordic countries or Nordic States or Nordic region or Scandinavia* or Denmark or Sweden or Finland or Norway).mp. [mp=title, abstract, heading word, drug trade name, original title, device manufacturer, drug manufacturer, device trade name, keyword, floating subheading word, candidate term word] (295217)

5 1 and 2 and 3 (340)

6 1 and 2 and 4 (482)

7 from 6 keep 12,16-18,26-27,30,63,69,77,117,125,128,132,187,193,207-208,237,245-246,252,255,260-261,264,279,286,315,330,332-333,338,340-341,352,358,361,364,368,370-371,380,387,391,395,406-407,409,417,421,426-429,436,439-440,444,447,452-453,460,462,464,467,471-472,474 (69)

8 from 5 keep 4,8,19,23-24,28,34,52,68,76,87,92,95,98,101,104,111,125,139,141,161-162,168,179,184,187,206,208,221,226-227,231,234,246,251-254,257-258,269,271,286,288,290-291,293,301-302,307,309-311,313,317,321,330,335-336,339 (60)

***************************

| **AFRICA-WIDE Search: Tuesday, June 26, 2018 7:46:21 AM** |
| --- |

| # | Query | Limiters/Expanders | Last Run Via | Results | Action |
| --- | --- | --- | --- | --- | --- |
| S3 | S1 AND S2 | Search modes - Boolean/Phrase | Interface - EBSCOhost Research Databases  Search Screen - Advanced Search  Database - Africa-Wide Information | 127 |  |
| S2 | Nigeria | Search modes - Boolean/Phrase | Interface - EBSCOhost Research Databases  Search Screen - Advanced Search  Database - Africa-Wide Information | 151,962 |  |
| S1 | cervical cancer screening | Search modes - Boolean/Phrase | Interface - EBSCOhost Research Databases  Search Screen - Advanced Search  Database - Africa-Wide Information | 744 |  |

**Database: Ovid MEDLINE(R) <2000 to June Week 4 2018>**

Search Strategy

1 (Cervical Cancer or Cervical Neoplasm* or Uterine cervical neoplasm* or Uterine cervical cancer* or Cancer of the uterine cervix or Cancer of cervix).mp. [mp=title, abstract, original title, name of substance word, subject heading word, floating sub-heading word, keyword heading word, protocol supplementary concept word, rare disease supplementary concept word, unique identifier, synonyms] (76323)

2 (Cervical Cancer or Cervical Neoplasm* or Uterine cervical neoplasm* or Uterine cervical cancer* or Cancer of the uterine cervix or Cancer of cervix).mp. [mp=title, abstract, original title, name of substance word, subject heading word, floating sub-heading word, keyword heading word, protocol supplementary concept word, rare disease supplementary concept word, unique identifier, synonyms] (76323)

3 (cancer screen* or Early cancer detection or Mass screening or Cancer screening test* or "Early diagnosis of cancer" or "Visual Inspection with Acetic Acid" or "HPV testing" or "HPV DNA Testing" or "Visual Inspection with Lugol Iodine" or VIA or VILI or "Papanicolaou’s smear" or Pap smear).mp. [mp=title, abstract, original title, name of substance word, subject heading word, floating sub-heading word, keyword heading word, protocol supplementary concept word, rare disease supplementary concept word, unique identifier, synonyms] (796338)

4 (Nordic countries or Nordic States or Nordic region or Scandinavia* or Denmark or Sweden or Finland or Norway).mp. [mp=title, abstract, original title, name of substance word, subject heading word, floating sub-heading word, keyword heading word, protocol supplementary concept word, rare disease supplementary concept word, unique identifier, synonyms] (215395)

5 1 and 3 and 4 (513)

6 from 5 keep 19,24,29,47,63,65,75,84-85,89,93,97,110,119,121,136,171,183-184,187-188,190,192,206,209,211,213,216,218,224-225,243,247,253,256,264,266,268,270,278-280,296-297,320,322-323,330,340-341,343,352-353,365,372,391,395,416,418,422,424,437,454,456-457,459,466,471 (68)

7 ("Nigeria" or "Africa, South of the Sahara" or "West* Africa" or "Federal Republic of Nigeria" or "Sub-Saharan Africa").mp. [mp=title, abstract, original title, name of substance word, subject heading word, floating sub-heading word, keyword heading word, protocol supplementary concept word, rare disease supplementary concept word, unique identifier, synonyms] (55994)

8 ("Nigeria" or "Africa, South of the Sahara" or "West* Africa" or "Federal Republic of Nigeria" or "Sub-Saharan Africa").mp. [mp=title, abstract, original title, name of substance word, subject heading word, floating sub-heading word, keyword heading word, protocol supplementary concept word, rare disease supplementary concept word, unique identifier, synonyms] (55994)

9 1 and 3 and 7 (194)

10 from 6 keep 1-68 (68)

11 from 9 keep 5,12,35,40,58-59,97-98,101-103,110,119,124,127,129,131,134,139,145,153-154,157,159,162,175,189 (27)

**CINAHL SEARCH: Thursday, July 26, 2018 7:39:51 AM**

| **#** | **Query** | **Limiters/Expanders** | **Last Run Via** | **Results** | **Action** |
| --- | --- | --- | --- | --- | --- |
| S6 | S1 AND S2 AND S5 | Search modes - Boolean/Phrase | Interface - EBSCOhost Research Databases  Search Screen - Advanced Search  Database - CINAHL Plus with Full Text | 82 |  |
| S5 | Nordic countries or Denmark or Finland or Norway or Sweden | Search modes - Boolean/Phrase | Interface - EBSCOhost Research Databases  Search Screen - Advanced Search  Database - CINAHL Plus with Full Text | 65,228 |  |
| S4 | S1 AND S2 AND S3 | Search modes - Boolean/Phrase | Interface - EBSCOhost Research Databases  Search Screen - Advanced Search  Database - CINAHL Plus with Full Text | 118 |  |
| S3 | Nigeria or Nigerian or Africa or sub-Saharan Africa | Search modes - Boolean/Phrase | Interface - EBSCOhost Research Databases  Search Screen - Advanced Search  Database - CINAHL Plus with Full Text | 41,710 |  |
| S2 | cervical cancer or cervical malignancy or cervical carcinoma | Search modes - Boolean/Phrase | Interface - EBSCOhost Research Databases  Search Screen - Advanced Search  Database - CINAHL Plus with Full Text | 9,080 |  |
| S1 | cervical cancer screening or pap smear or cervical smears | Search modes - Boolean/Phrase | Interface - EBSCOhost Research Databases  Search Screen - Advanced Search  Database - CINAHL Plus with Full Text | 7,083 |  |

Loading...

Top of Form

**APPENDIX 4: CRITICAL APPRAISAL TOOLS**

For Randomized Interventions and Descriptive Studies, the NIH QAT for controlled interventions and NIH QAT for Observational Cohort and Cross-Sectional Studies were used.

It uses a ratings system to judge each study to be of "good," "fair," or "poor" quality. The ratings on the different items were used to assess the risk of bias in the study due to flaws in study design or implementation.

The questionnaires applied are NIH QAT for controlled interventions and NIH QAT for Observational Cohort or Cross-Sectional Studies [here](https://www.nhlbi.nih.gov/health-topics/study-quality-assessment-tools).

General Guidance for determining the overall Quality Rating of the studies were found [here](https://www.nhlbi.nih.gov/node/80102).

Key ideas to note are that the questions on the assessment tool were designed to help reviewers focus on the key concepts for evaluating a study's internal validity. They are not intended to create a list that is simply tallied up to arrive at a summary judgment of quality.

Internal validity is the extent to which the results (effects) reported in a study can truly be attributed to the intervention being evaluated and not to flaws in the design or conduct of the study–in other words, the ability for the study to make causal conclusions about the effects of the intervention being tested. Such flaws can increase the risk of bias. Critical appraisal involves considering the risk of potential for allocation bias, measurement bias, or confounding (the mixture of exposures that one cannot tease out from each other). Examples of confounding include co-interventions, differences at baseline in patient characteristics, and other issues addressed in the questions above. High risk of bias translates to a rating of poor quality. Low risk of bias translates to a rating of good quality.

Fatal flaws: If a study has a "fatal flaw," then risk of bias is significant, and the study is of poor quality. Examples of fatal flaws in RCTs include high dropout rates, high differential dropout rates, no ITT analysis or other unsuitable statistical analysis (e.g., completers-only analysis).

**APPENDIX 5: CAAP TABLE FOR GREY LITERATURE**

| AUTHOR | TITLE | CURRENCY | AUTHORITY | ACCURACY | PURPOSE |
| --- | --- | --- | --- | --- | --- |
| African Population and Health Research Centre; International Agency for Research on Cancer; World Health Organization; 2012^16^ | Prevention of cervical cancer through screening using visual inspection with acetic acid (VIA) and treatment with cryotherapy | Current | Obtained from The World Health Organization/Also available at IARC Website | No inconsistencies; limitations were clearly stated | For global health and health policy professionals |
| World Health Organization; May 2007^17^ | Strengthening cervical cancer prevention in Europe: Meeting of policymakers and programme managers (Meeting Report) | Current | Obtained from The World Health Organization | No inconsistencies noted | Meeting minutes and recommendations are for global health and health policy professionals |

**APPENDIX 6: SAMPLE OF DATA EXTRACTION FORM**

Author; Date: Bassey, Emem E; Ekpo, Mmefin D; Abasiattai, Aniekan M; Ekanem, Mabel I; April 2008

Title of publication: Cervical Cancer Screening in Uyo, South-South Nigeria

Country/countries of study: Nigeria

Aim of the study: Reviewed the cervical cancer screening exercise of the Medical Women Association of Nigeria which lasted between 1996-2011

Study design: Quantitative Study

Types of interventions (and methods): Pap Smear

Duration of the intervention: Monthly screenings between June 1996 to July 2001

Main outcomes: Only 332 women participated in the 5yrs of the free monthly screening

Outcomes measured at population or individual level: The incidence of CIN in this study was 12%, which was like 11.8% reported in Ibadan and 12.2% of abnormal smears in Enugu.

Main results: Majority 209 (75.7%) were negative smears, followed by inflammatory changes/chronic cervicitis (10.5%). Cervical intraepithelial neoplasia (CIN) was diagnosed in 33 smears (12%) and 1 smear (0.36%) was suspicious for invasive cancer which was confirmed by biopsy.

Key conclusions of the study authors: “Although there are facilities for cytology in some Nigerian hospitals, these serve only a limited number of women. The only other screening exercises are carried out by non- governmental bodies such as the Medical Women's Association of Nigeria (MWAN) and such exercises are usually limited by poor funding, inadequate logistics and lack of public awareness. Even among female health professionals, despite being highly aware very few of them have ever had a cervical smear done”

Biases identified by authors: Not Specified

Additional notes by reviewer

Checklist used: NIH QAT

Strengths: Long study period; similar prevalence to studies carried out in other locations

Weaknesses: limited information, few study participants, unable to ascertain if biases and confounding factors were sufficiently considered, ethical consideration not stated

Summary of appraisal findings: Low quality but will be included (This will be taken into consideration in the emphasis it receives in the discussion section)

**APPENDIX 7: DATA EXTRACTION WITH CRITICAL APPRAISAL**

| **AUTHOR** | **DATE OF PUBLICATION** | **TITLE** | **TYPE OF PUBLICATION** | **COUNTRY** | **OUTCOMES** | **CONCLUSION** | **CRITICAL APPRAISAL^^[[1]](#footnote-1)^^** |
| --- | --- | --- | --- | --- | --- | --- | --- |
| **DESCRIPTIVE PAPERS (NIGERIA)** | | | | | | | |
| Bassey et al^29^ | Apr-08 | Cervical Cancer Screening in Uyo, South-South Nigeria | Journal Article/Descriptive study | Nigeria | The study reports poor participation of the target population as only 332 women participated in the 5yrs when free monthly screening was offered. | There are no organized cervical cancer screening programmes though there are facilities for cytology in some Nigerian hospitals which serve limited number of women. | Low |
| Chukwuali et al^30^ | Oct-03 | Cervical Cancer Screening in Enugu | Descriptive Study/ Journal Article | Nigeria | The study reports that only 815 women participated in the highly subsidized screening service in Enugu over a 10yr period | Due to reasons such as poor awareness and socio-cultural barriers, subsidized cervical cancer screening was not adequately utilized. | Fair |
| Adepoju et al^31^ | Aug-16 | Targeting women with free cervical cancer screening: Challenges and lessons learnt from Osun State, Southwest Nigeria. | Journal Article/Descriptive study | Nigeria | The study identified the two main challenges to accessing cervical cancer screening: low risk perception and logistical issues in rural areas | They concluded that since most participants were urban based, there is need to decentralize cancer of cervix screening through mobile clinics and establishment of screening centers in the rural areas. | Fair |
| Obi et al^32^ | Apr-07 | Participation in highly subsidized cervical cancer screening by women in Enugu, South-east Nigeria. | Journal Article/Descriptive study | Nigeria | One of the findings was poor participation as<1% (932 women) of target population participated | Their conclusion was to it was not enough to provide cervical cancer screening services but there is need to follow up these services by sustained awareness campaigns and motivation of health care providers to offer appropriate information to patients. | Low |
| Nnadi et al^33^ | Mar-16 | Screening for Cervical Cancer: Experience from a University Hospital in North Western Nigeria (2007 ‑ 2009) | Journal Article/Descriptive study | Nigeria | Main outcomes indicated that participation was extremely poor compared to similar studies done in other parts of the country (routine cervical smear was performed in only 20.6% of the subjects in this study compared to 53.8% in a similar study in Ibadan). It was also noted that indication for cervical screening was mostly symptom based | The authors concluded that only through formulation and implementation of an organized national screening programme (while maximizing opportunistic screening in the interim) can screening be done more effectively and efficiently. | Good |
| **RANDOMIZED INTERVENTIONS (NIGERIA)** | | | | | | | |
| Okeke et al^34^ | Oct-12 | What is the price of prevention? New evidence from a field experiment. | Journal Article/Randomized Experimental study | Nigeria | One of the outcomes was that the women who were randomly selected to receive the conditional cancer treatment subsidy were about 4% more likely to accept cervical cancer screening | The authors concluded that the optimal set of subsidies should include treatment subsidies (if the client is screened positive) in addition to screening price subsidies | Good |
| **RANDOMIZED CONTROLLED TRIALS (NORDIC COUNTRIES)** | | | | | | | |
| Alfonzo et al^35^ | Mar-16 | Effect of Fee on Cervical Cancer Screening Attendance--ScreenFee, a Swedish Population-Based Randomized Trial. | Journal Article/Population-based Randomized Controlled Trial | Sweden | The main outcome of this study was that participation was not affected by the absence or presence of a fee. | They concluded that other strategies could be employed in socially disadvantaged urban districts as abolishing fees did not increase attendance in the short term. | Good |
| Jensen et al^36^ | Feb-09 | A primary healthcare-based intervention to improve a Danish cervical cancer screening programme: A cluster randomized controlled trial. | Journal Article/Cluster Randomized Controlled trial | Denmark | The outcome included improved participation and improved coverage when women were targeted with invitations and improving GPs’ attention to cervical cancer programmes. | Their conclusion was that using a special targeted invitation to non-attendees combined with increasing general practitioners’ attention to the programme could improve women’s participation and increase coverage of cervical cancer screening | Good |
| Elfstrom et al^37^ | Jan-16 | Registry-based assessment of the status of cervical screening in Sweden. | Population based descriptive study**/**Journal Article | Sweden | They noted that analyzing key quality indicators forms the basis for quality improvement of the organized cervical screening programme. | They concluded that regular registry-based monitoring and evaluation of QI indicators can provide evidence base for prioritization of improvement strategies. | Good |
| **COHORT STUDY** | | | | | | | |
| Vaccarella et al^38^ | Jul-16 | 50 years of screening in the Nordic countries: Quantifying the effects on cervical cancer incidence. | Journal Article/Cohort Study | Denmark, Finland, Norway and Sweden | The authors projected that in the absence of screening, incidence rates for 2006–2010 in Nordic countries would have been -5 times higher than observed rates. | They conclude that the organized screening programmes in these countries has resulted in the low incidence of cervical cancer in these four Nordic countries. | Good |
| **REVIEW ARTICLES** | | | | | | | |
| Dillner^39^ | Aug-00 | Cervical cancer screening in Sweden. | Review Article | Sweden | They noted that cervical cancer screening in Sweden is heterogeneous in quality i.e. some counties practice organized screening and others are opportunistic. Hence only 950 000 Papanicolaou (Pap) smears (31%) are taken annually in the organized screening programme. | They conclude that more studies need to be done to assess the effect of organized screening vs spontaneous screening on cervical cancer mortality. | Good |
| Anttila et al^40^ | Aug-00 | Cervical cancer screening programme in Finland. | Review Article | Finland | The article notes that incidence of cervical cancer has decreased in Finland and attributes it to organized screening activities. | The authors conclude that the 30yr-old organized screening programme has resulted in a decrease of over 70% cervical cancer incidence and a reduction in cervical cancer mortality. | Good |
| Bigaard et al^41^ | Aug-00 | Cervical cancer screening in Denmark. | Review Article | Denmark | This review noted that the Danish screening programme had good coverage as a total of 650 000 smears were taken annually which corresponds to screening of all Danish women aged 25-59 years on average, every second year even though the guidelines recommend screening every 3^rd^ year. They also highlighted a decrease in incidence from 15.3 per 100 000 women in 1987- 1992 to 12.9 per 100 000 women in 1993-1995. | The authors conclude that organized screening has a better preventive effect than opportunistic screening; they recommend a move towards a longer screening interval than the 3-year interval currently practiced. | Good |
| **GREY LITERATURE** | | | | | | | |
| African Population and Health Research Centre; International Agency for Research on Cancer; World Health Organization; 2012^42^ | | Prevention of cervical cancer through screening using visual inspection with acetic acid (VIA) and treatment with cryotherapy | | Demonstration Project across 6 countries: Madagascar, Malawi, Nigeria, Uganda, the United Republic of Tanzania and Zambia | The reports conclude that “screen and treat” approach can be used in low-resource countries. Results indicated that it is acceptable and feasible at low-level health facilities in these six African countries. | | |
| World Health Organization; May 2007^43^ | | Strengthening cervical cancer prevention in Europe: Meeting of policymakers and programme managers  (Meeting Report) | | WHO European Region | The report used the Finnish cervical cancer screening programme to exemplify a cost-effective way to run an organized programme. This is because the Finnish programme has proven to be very effective in reducing the incidence of and mortality from cervical cancer by screening a narrow target group (30–60 years) with a wide screening interval (5 years)  The report also highlighted VIA as a viable option to pap smears in low to middle income countries as evidence shows that it is feasible and effective in areas where access is difficult. | | |

1. Based on the NIH QAT^27^ [↑](#footnote-ref-1)
